# Massive seasonal high-altitude migrations of nocturnal insects above the agricultural plains of East China

**DOI:** 10.1073/pnas.2317646121

**Published:** 2024-04-22

**Authors:** Jianrong Huang, Hongqiang Feng, V. Alistair Drake, Don R. Reynolds, Boya Gao, Fajun Chen, Guoyan Zhang, Junsheng Zhu, Yuebo Gao, Baoping Zhai, Guoping Li, Caihong Tian, Bo Huang, Gao Hu, Jason W. Chapman

**Affiliations:** ^a^Henan Key Laboratory of Crop Pest Control, Key Laboratory for Integrated Crop Pests Management on Crops in Southern Region of North China, International Joint Research Laboratory for Crop Protection of Henan, No. 0 Entomological Radar Field Scientific Observation and Research Station of Henan Province, Institute of Plant Protection, Henan Academy of Agricultural Sciences, Zhengzhou, Henan 450002, China; ^b^Centre for Ecology and Conservation, and Environment and Sustainability Institute, University of Exeter, Penryn, Cornwall TR10 9FE, United Kingdom; ^c^School of Science, UNSW Canberra, The University of New South Wales, Canberra, ACT 2610, Australia; ^d^Institute for Applied Ecology, Faculty of Science and Technology, University of Canberra, Canberra, ACT 2617, Australia; ^e^Natural Resources Institute, University of Greenwich, Chatham, Kent ME4 4 TB, United Kingdom; ^f^Department of Computational and Analytical Sciences, Rothamsted Research, Harpenden, Herts AL5 2JQ, United Kingdom; ^g^Department of Entomology, College of Plant Protection, Nanjing Agricultural University, Nanjing, Jiangsu 210095, China; ^h^Plant Protection and Quarantine Station of Henan Province, Zhengzhou, Henan 450002, China; ^i^Shandong Agricultural Technology Extension Center, Jinan, Shandong 250100, China; ^j^Institute of Plant Protection, Jilin Academy of Agricultural Sciences, Gongzhuling, Jilin 136100, China

**Keywords:** insect migration, radar entomology, Lepidoptera, biomass flux, crop pests

## Abstract

Long-distance migrations of insects contribute to ecosystem functioning but also have important economic impacts when the migrants are pests or provide ecosystem services. We combined radar monitoring, aerial sampling, and searchlight trapping, to quantify the annual pattern of nocturnal insect migration above the densely populated agricultural lands of East China. A total of ~9.3 trillion nocturnal insect migrants (15,000 t of biomass), predominantly Lepidoptera, Hemiptera, and Diptera, including many crop pests and disease vectors, fly at heights up to 1 km above this 600 km-wide region every year. Larger migrants (>10 mg) exhibited seasonal reversal of movement directions, comprising northward expansion during spring and summer, followed by southward movements during fall. This north–south transfer was not balanced, however, with southward movement in fall 0.66× that of northward movement in spring and summer. Spring and summer migrations were strongest when the wind had a northward component, while in fall, stronger movements occurred on winds that allowed movement with a southward component; heading directions of larger insects were generally close to the track direction. These findings indicate adaptations leading to movement in seasonally favorable directions. We compare our results from China with similar studies in Europe and North America and conclude that ecological patterns and behavioral adaptations are similar across the Northern Hemisphere. The predominance of pests among these nocturnal migrants has severe implications for food security and grower prosperity throughout this heavily populated region, and knowledge of their migrations is potentially valuable for forecasting pest impacts and planning timely management actions.

## Significance

High-altitude, windborne movements of insects occur on an enormous scale, and have significant impacts on ecosystem function, provision of beneficial services, disease spread, and agricultural productivity. We used a combination of insect monitoring radar, balloon-borne nets, and searchlight traps to characterize the intensity, taxonomic composition, direction, and geographical extent of nocturnal insect “bioflows” occurring at heights to ~1 km above the agricultural lands of East China during spring, summer, and fall. We demonstrate seasonal northward and southward flows and show that the transport of insect biomass is considerably greater above this globally important food-production region than above the United Kingdom (the only other region where it has been quantified to date) and is dominated by species that are agricultural pests.

Seasonal insect migrations lead to huge “bioflows” in the lower atmosphere (i.e., up to ~2 km above ground) over the temperate and subtropical regions of the Earth. These long-range mass movements impact ecosystem function through food web interactions, nutrient and energy transfer, and vectoring of propagules, parasites, and pathogens between distant ecosystems (1–7). Migrant insects can be beneficial, providing valuable ecosystem services for society such as biocontrol and pollination (7–10), but many are pests, causing harm by directly damaging crops and spreading plant, animal, and human diseases (10–14). There is increasing evidence that beneficial insects, particularly pollinators, are in decline (15–17) and that insect pest impacts are becoming more burdensome (18, 19).

The first step in determining the ecological and economic significance of insect bioflows is to quantify them. High-altitude migratory movements (i.e., movements well above the vegetation canopy, typically at heights of a few hundred meters) have customarily been investigated by aerial sampling, which is effective for the more numerous small insects (<10 mg) (20–23). However, the much lower densities typical for larger insects greatly reduce their probability of capture; fortunately, this fraction of the bioflow is amenable to study by special-purpose entomological radars (24, 25). Since the late 1990s, insect monitoring radars (IMRs) (26) have operated autonomously in Australia and the United Kingdom; these provide important behavioral and ecological data on individual high-flying insect migrants, such as flight altitudes, windborne displacement vectors, and self-powered flight headings, and from these values, measures of migration intensity such as traffic rates, total overflights and biomass transfers can be estimated (5, 9) and used to support operational pest forecasting (6, 27, 28). Datasets obtained from IMR networks operated for long periods (~10 y) have resulted in an understanding of the orientation mechanisms and behavioral strategies adopted by high-flying insect migrants (29–32). They have also enabled quantification of the impact of insect migrations on ecosystem function and provision of ecosystem services, and elucidation of the migration patterns of a range of species and of the geographical and climate factors that drive migration (5, 9, 33).

**Fig. 1.**
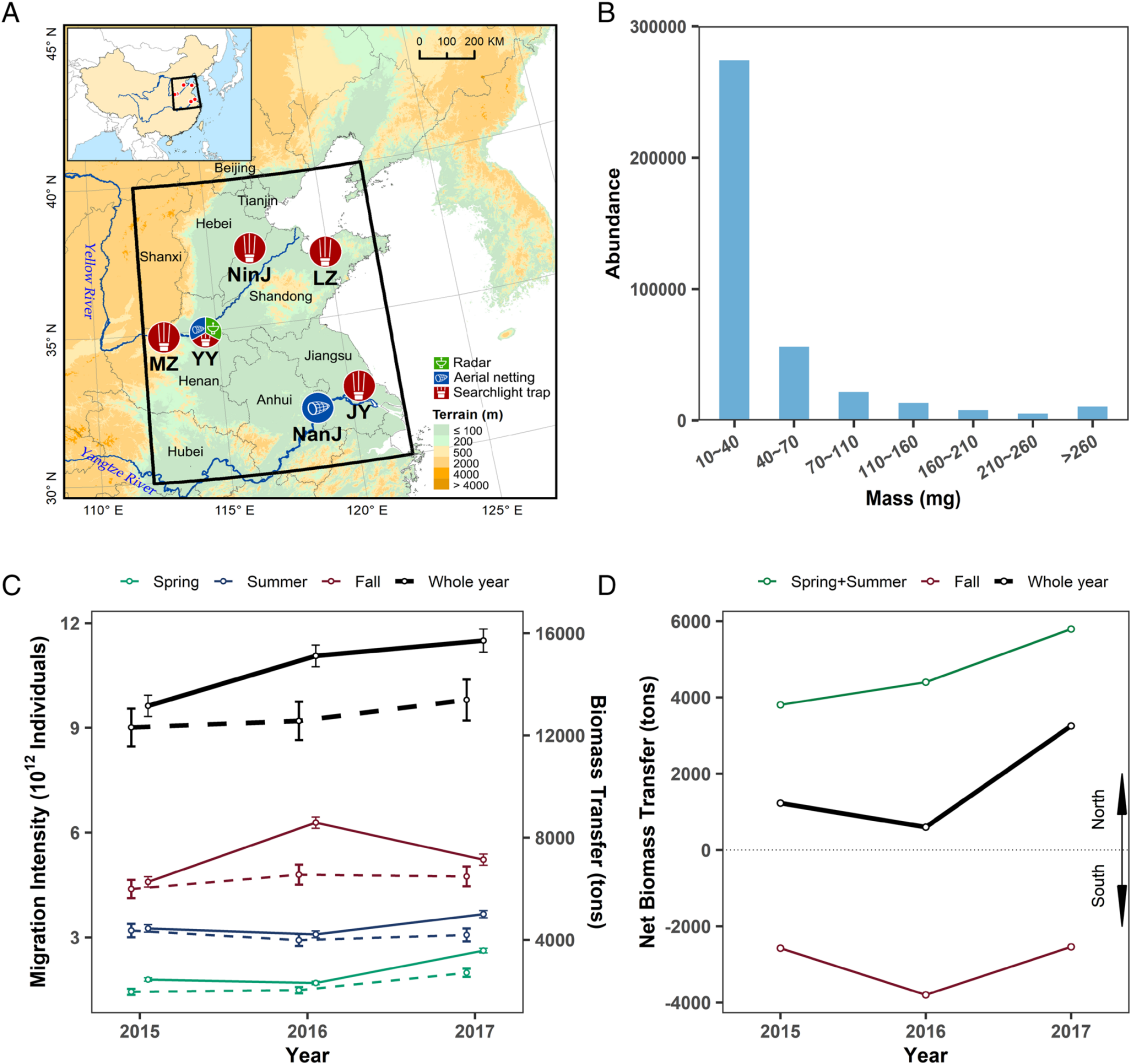
Migration arena of the ECP. (*A*) Map of East China, with the ECP region (black outline), major rivers and provinces indicated. The IMR and a searchlight trap were operated in Yuanyang County (YY) of Henan province; additional searchlight traps were operated at: Mengzhou City (MZ), Henan; Ningjin County (NinJ), Shandong; Laizhou City (LZ), Shandong; and Jiangyan District (JY), Taizhou City, Jiangsu. Aerial faunas were sampled at ~200 m by balloon-supported aerial netting at YY and near Nanjing (NanJ), Jiangsu. (*B*) Frequency distribution of estimated masses of IMR-detected (i.e., “larger”) nocturnal insects for the full 2015 to 2017 observation period. (*C*) Year-to-year variation of annual abundance (in trillions of individuals) and biomass transfer (in tons) of all nocturnal insects migrating above a 600-km-diameter region of the ECP, estimated from IMR observations and aerial-trapping data (*SI Appendix*, Tables S8 and S9). Solid lines show the biomass transfer, dashed lines the migration intensity; the points show the values calculated with the mean ratio of small insects/larger insects (38.4:1) and the error bars show the values when the two single-year ratios (40.67:1 and 36.04:1) are used. (*D*) Annual patterns of net directional biomass transfer of larger nocturnal insects migrating northward (positive values) and southward (negative) across the ECP (*SI Appendix*, Table S11).

Entomological radar studies have been carried out in China since the 1980s, confirming a general pattern of windborne northward movement in spring, breeding at higher latitudes over one or more generations, and a southward return movement in fall (34–41). However, these research programs used scanning entomological radars, which depend on manual operation and were therefore not suitable for continuous long-term monitoring. More recently, automated vertical-looking IMRs (24–26), capable of obtaining datasets extending over multiple years, have been deployed in East China and operated in conjunction with aerial netting and a network of upward-pointing searchlight traps to support target identification (42). The study area extends over the North China Plain and the adjoining Middle and Lower Yangtze River regions (Fig. 1*A*), which in combination we hereafter term the East China Plain (ECP). Extending 600 km from east to west and 1,000 km from north to south (10), this is one of the most densely populated agricultural areas in the world and the biggest food production region of China (43). Insect movements here are part of a more extensive insect migration “flyway” [a term we adopt from ornithology (44)], extending as far south as Indochina (45), and north to Northeast China, the Korean Peninsula, and Japan (46, 47); within this region, the movement of insect pests, disease vectors, and beneficials affects crop yields and grower livelihoods on a massive scale (48). In this paper, we draw on IMR and trapping data to quantify seasonal migration patterns of nocturnal high-flying insects, and the associated biomass and nutrient transfers, over the ECP. We compare our estimates with those from the only similar research program, undertaken in the United Kingdom (2–5, 9), and identify biodiversity, climate, and land-use factors that might account for differences in migration patterns between these two temperate-climate cropping regions.

## Results and Discussion

### Abundance and Biomass Transfers.

We estimated the seasonal and annual abundances of nocturnal high-flying insects migrating above the ECP at heights of 100 to 1,800 m from three data sources: an IMR located at Yuanyang near Zhengzhou, Henan, in the central ECP; five searchlight traps distributed across the ECP; and aerial netting catches at two locations in the ECP (Fig. 1*A*). The estimates were made separately for three contiguous size categories, denoted here as “small” (<10 mg), “medium” (10 to 70 mg), and “large” (70 to 500 mg); in some analyses, the last two categories are combined and referred to collectively as “larger” insects. Values for larger insects (Fig. 1*B*) were extrapolated from IMR observations of 388,233 beam-transiting individuals (*SI Appendix*, Table S1) and from catches of larger insects in the searchlight traps (*SI Appendix*, Figs. S1 and S2 and Tables S2 and S3). The small insects produce echoes that are too weak to be efficiently detected by IMRs, so we used catches in aerial nets (*SI Appendix*, Fig. S3 and Tables S4–S7) to estimate the relative proportion of small insects to larger insects in the nocturnal migrant population (*SI Appendix*, Table S6) and then used this factor to estimate the abundance of the entire insect population above the night-time ECP.

Employing these methods, we find that annual mean nocturnal migratory overflights of all size categories combined (and in all directions) were 15.5 billion insects per kilometer (range over the 3 y: 15.0 to 16.3 × 10^9^ km^−1^), comprising 97.5% small insects, 2.4% medium, and 0.2% large; taking account of the insect masses (*SI Appendix*, Tables S2–S5), there were annual omnidirectional biomass transfers of 24.4 t km^−1^ (range over the 3 y: 21.9 to 26.1 t km^−1^), of which small insects accounted for 47.8% (*SI Appendix*, Tables S8 and S9). To determine whether these values could be scaled up to the full 600 km width of the ECP, we examined the combined abundance of three noctuid moth species (*Agrotis ipsilon*, *Helicoverpa armigera*, and *Mythimna separata*; all important pests of ECP crops) in the five searchlight traps. While species composition between sites did vary significantly (ANOVA; *F*_2,35_ = 6.55, *P* = 0.004; *SI Appendix*, Fig. S1), there was no difference in the total combined abundance of the three species with location (ANOVA; *F*_4,35_ = 1.54, *P* = 0.213, *SI Appendix*, Fig. S1) or year (ANOVA; *F*_2,35_ = 1.67, *P* = 0.203). We therefore inferred that migration intensity was relatively consistent across the region and scaled our previous estimates to produce values for region-wide migrant abundance and biomass transfer. As these are omnidirectional flows, the estimates are for transfers within a notional circular area with a diameter of ~600 km located at the latitude of the IMR (i.e., 35°N). Our results indicated that a mean of 9.3 trillion (annual range 9.0 to 9.8 × 10^12^) nocturnal insects migrated within this area each year, producing a mean combined omnidirectional biomass transfer of 14,600 t (range: 13,100 to 15,600 t; Fig. 1*C* and *SI Appendix*, Tables S8 and S9). Although the larger insects only comprised 2.54% of the numerical total (Fig. 1*B*), the numbers were still substantial: 214 to 230 billion medium insects and 11 to 20 billion large insects (*SI Appendix*, Table S8). Due to their size, these larger insects contributed a much greater proportion of the biomass transfer, 48.6 to 54.3% of the annual totals (*SI Appendix*, Table S9).

### Composition and Pest Status of the Aerial Fauna.

We made a detailed analysis of the catches in the searchlight trap located closest to the IMR, to assess the taxonomic composition of the larger species likely to be responsible for the radar-detected insects (*SI Appendix*, Figs. S1 and S2 and Tables S2 and S3). The Lepidoptera were the most dominant group, accounting for 39.8% of all searchlight-trap catches; however, after exclusion of chafer beetles (Coleoptera; Scarabaeidae), crickets (Orthoptera; Gryllidae), and a few other minor groups that were all assessed as unlikely to be *high-flying* migrants (*Methods*), the Lepidoptera proportion rose to 76.2% of the nocturnal migrant sample (*SI Appendix*, Fig. S2 and Table S2). Of the 122 species of larger-sized Lepidoptera (>10 mg) caught in the searchlight trap, 80 (66%) are considered pests (*SI Appendix*, Table S3), with all 10 most abundant species being important pests of field crops, or (in one case) ornamental trees, and well known for their long-range migrations. These were *A. ipsilon*, *Athetis lepigone*, *H. armigera*, *Leucania loreyi*, *M. separata,* and *Spodoptera exigua* (all Noctuidae); and *Botyodes diniasalis*, *Conogethes punctiferalis*, *Ostrinia nubilalis,* and *Spoladea recurvalis* (all Crambidae) (10, 36–42, 47). Thus, we infer that the majority of the radar-detected larger nocturnal insect migrants were pest Lepidoptera (*SI Appendix*, Figs. S1 and S2), predominantly members of the Noctuidae in the large category and Crambidae in the medium category.

Pest insects were also important components of the small-insect fauna sampled by aerial netting above the ECP (*SI Appendix*, Fig. S3). Sucking hemipteran crop pests (families Delphacidae, Cicadellidae, and Aphididae), which cause physical damage and act as vectors of important crop diseases [e.g., rice black-streaked dwarf virus, maize rough dwarf virus, and barley yellow dwarf virus (11, 45, 49, 50)], comprised 39.3% of the aerial samples of small insects (*SI Appendix*, Fig. S3 and Table S7). The most abundant of these were rice planthoppers (*SI Appendix*, Table S4), the most important rice pests globally and well-known long-distance migrants (14, 35, 45, 51, 52). The other major component of the aerial samples (comprising 35.6% of the aerial fauna) were unidentified minute Diptera (*SI Appendix*, Fig. S3 and Tables S4 and S7), from families such as Cecidomyiidae, Ceratopogonidae, Chironomidae, Culicidae, and Phoridae. Many cecid midges are crop pests, while mosquitoes and biting midges are migrant vectors of animal and human diseases (12, 13, 53–55), although the principal ecological role of these minute Diptera is likely to be as superabundant prey items of nocturnal insectivores. In combination, the searchlight trapping and aerial sampling show that the migrant nocturnal fauna consists principally of Lepidoptera, Hemiptera, and Diptera and that agricultural crop pests and disease vectors predominate.

### Altitude Selection and Ground Speed.

**Fig. 2.**
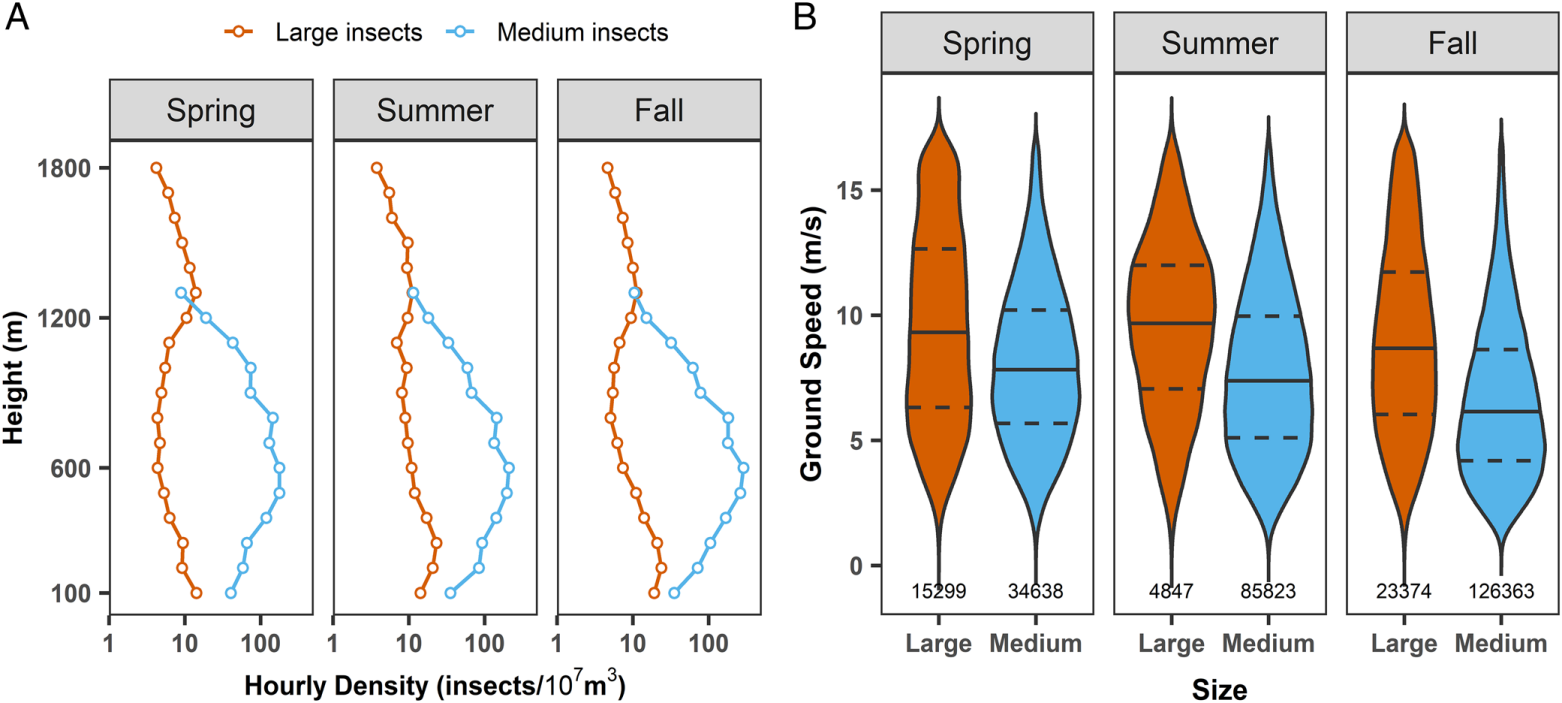
Height of flight and ground speed of larger nocturnal insect migrants. (*A*) Vertical profiles (above 100 m) of the aerial density recorded by the IMR during mass migrations of large (brown) and medium (blue) insects in each season. (*B*) Ground speed during insect mass migrations of large and medium insects. Large insects traveled significantly faster than medium insects in each season (mean track speed ± SD; spring: 9.5 ± 3.9 vs. 8.1 ± 3.1 m s^−1^, *t* = 33.44, *P* < 0.0001; summer: 9.6 ± 3.3 vs. 7.7 ± 3.3 m s^−1^, *t* = 36.61, *P* < 0.0001; fall: 9.0 ± 3.7 vs. 6.6 ± 3.1 m s^−1^, *t* = 79.44, *P* < 0.0001). In the violin plots, solid lines represent the median value, dashed lines represent the 1st and 3rd quartile value, and the shape contour indicates the density of data points along the range of speeds; the number below the violin is the sample size.

Both size categories of larger nocturnal migrants showed considerable vertical stratification in their aerial density profiles (Fig. 2*A*). Large insect migration intensity was highest at the lowest measured flight altitudes in each season, about 100 to 300 m above ground level; medium insect intensity peaked higher, at about 400 to 600 m above the ground (Fig. 2*A*). Similar patterns of nocturnal altitudinal layering have previously been observed in radar studies from many regions (24, 25), including China (36–40, 56), where density maxima at heights of a few hundred meters are typically found in warm, fast-moving airstreams associated with the formation of low-level nocturnal jets (57). There was some evidence for this above the ECP, as winds in the study period were slightly faster around 500 m than at other heights (*SI Appendix*, Fig. S4*A*), and night-time air temperatures at this height were comparatively warm with median values around 20 °C in spring (April and May), 28 °C in summer (June and July), and 25 °C in fall (August to October) (*SI Appendix*, Fig. S4*B*). Adaptive flight-altitude selection as seen here can lead to rapid windborne displacement and long flight durations (and hence long-distance transport), due to the warm and fast-moving airstreams often found at the preferred flight height. Winds experienced during “mass migrations” (occasions on which most migration occurred; see *Methods*) above the ECP were, however, comparatively slow, typically only 5 to 7 m s^−1^ (and exceeding 10 m s^−1^ only infrequently; *SI Appendix*, Fig. S5). The IMR-observed ground speeds were typically 3 to 11 m s^−1^ (11 to 40 km h^−1^) for the medium insects, and significantly faster at 5 to 13 m s^−1^ (18 to 47 km h^−1^) for the large insects (Fig. 2*B* and *SI Appendix*, Fig. S6), these values being consistent with the combined effect of wind and airspeed if the larger insects’ self-powered airspeeds are 1 to 3 m s^−1^ faster than those of the medium insects. The observed ground speeds are 2 to 4× faster than the migrant insects would be able to travel under their self-powered flight alone, indicating the importance of wind transport for long-distance travel. At such speeds, 8 h of nocturnal migration would result in windborne displacements of 200 to 300 km per night, depending on the wind speed at flight height and the migrants’ body size.

### Seasonal Migration Directions, Orientation Behavior, and Wind Selectivity.

**Fig. 3.**
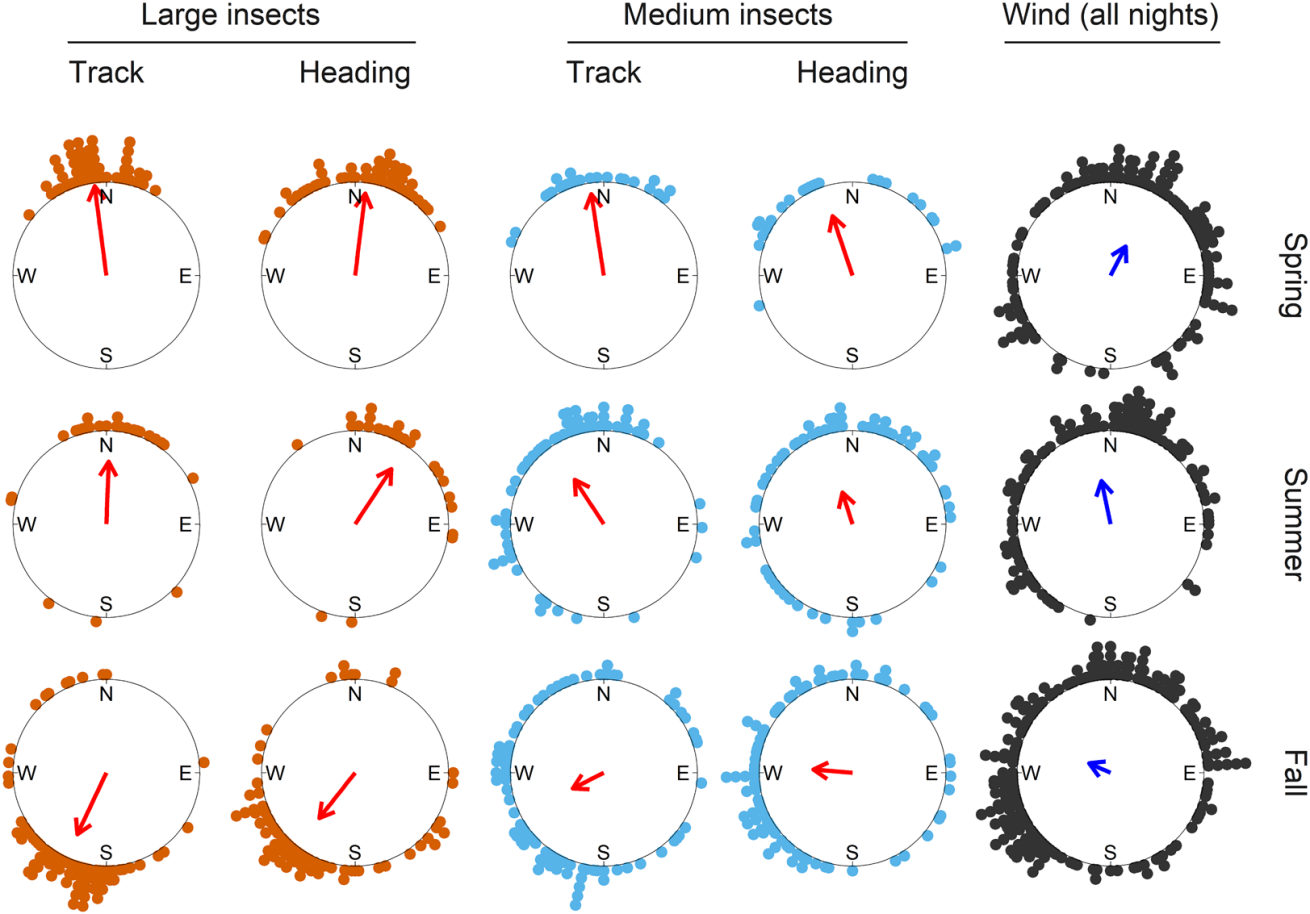
Seasonal migration directions and flight headings of larger nocturnal insect migrants during mass-migration events, and downwind directions on all nights. Colored dots on the periphery of the circles represent the mean track, heading, or downwind direction on each night. The bearing of the red and blue arrows indicates the seasonal mean direction, and arrow length represents the circular resultant length (*r*), a measure of the clustering of the data around the mean. Full circular statistics are presented in *SI Appendix*, Table S10. Insect data are from the IMR; winds are from the ECMWF reanalysis.

**Fig. 4.**
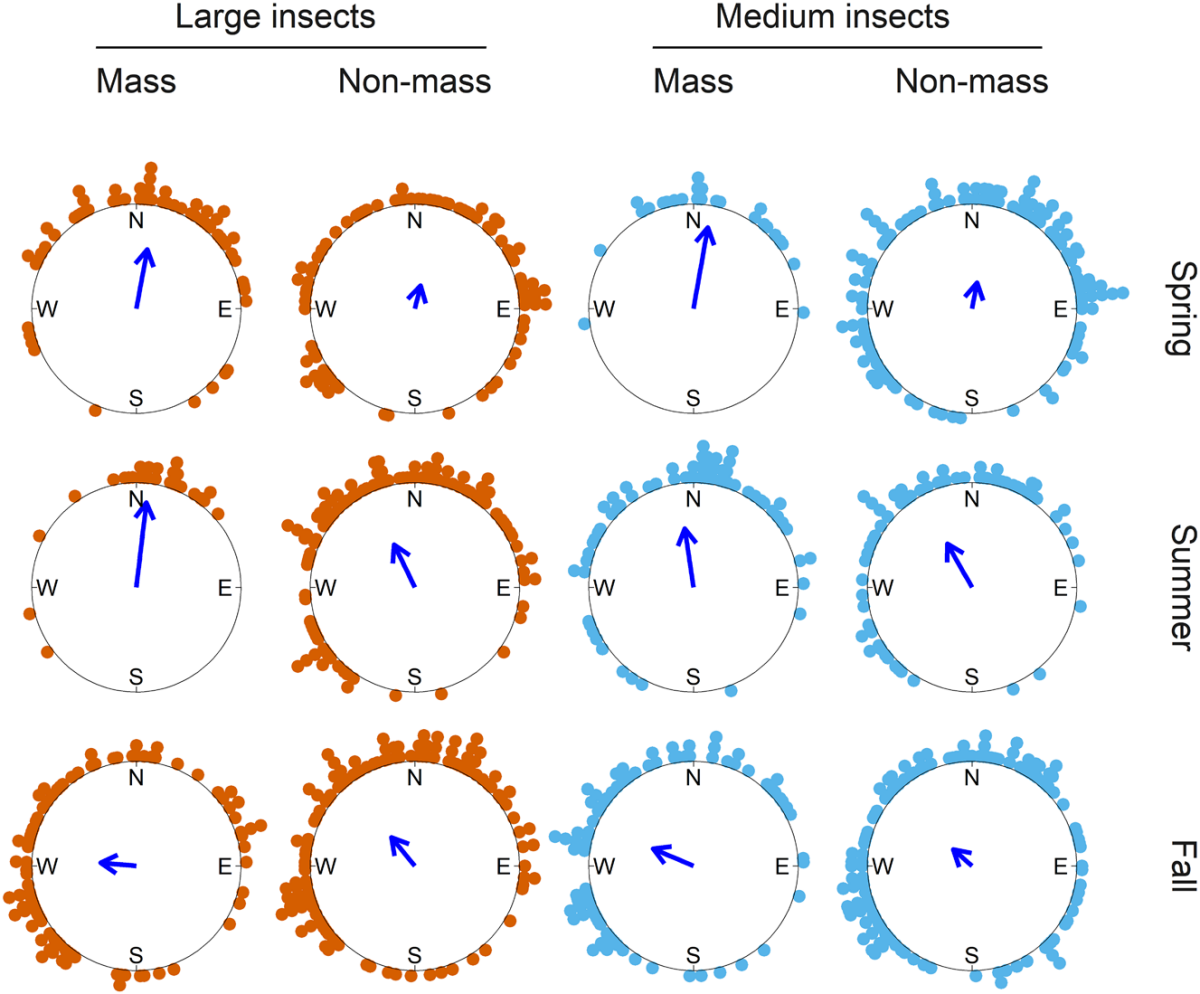
Seasonal patterns of downwind directions during mass migration nights and nonmass migration nights for larger insects. Colored dots on the periphery of the circles represent mean downwind directions on each night. The bearing of the blue arrows indicates the seasonal mean downwind direction, and arrow length represents the circular resultant length (*r*), a measure of the clustering of the data around the mean. Full circular statistics are presented in *SI Appendix*, Table S10. Mass/nonmass classification is from IMR data; winds are from ECMWF reanalysis.

We compared directional data measured by the IMR from 178 mass migrations of the medium nocturnal migrants (involving 246,824 individual insects) and the large migrants (involving 43,520 individuals; *SI Appendix*, Table S1), during spring, summer, and fall with wind data from reanalyzed meteorological observations. Downwind directions at migration heights on all nights had mean values toward north in spring and summer and northwest in fall (Rayleigh tests; spring mean downwind direction: 28°, summer: 348°, fall: 294°; Fig. 3 and *SI Appendix*, Table S10). Mass migration directions, however, of both size categories of migrants showed a clear seasonal reversal of direction. Migration tracks were toward the north or northwest during spring (large: 353°, medium: 351°) and summer (large: 2°, medium: 327°; Fig. 3 and *SI Appendix*, Table S10), with this northward transport facilitated by the broadly favorable southerly winds. However, there was evidence of wind selectivity during spring and summer: when comparing mass migration nights with nights with sparse migration, clustering of wind directions around the overall (northward-pointing) means was tighter during mass migrations (as indicated by the larger *r*-values; Fig. 4 and *SI Appendix*, Table S10). Thus, there is evidence that spring migrants chose to fly on nights with the most favorably directed winds (i.e., winds blowing toward the north). Similar patterns of more intense movement on winds blowing in favorable directions during spring have been observed in many high-altitude mass insect migrations in the United Kingdom (2, 5, 29, 32).

During fall, there was clear evidence that migrants migrated preferentially back toward the winter-breeding regions, as movements were principally toward the southwest in both size categories (large: 205°, medium: 243°; Fig. 3 and *SI Appendix*, Table S10). This was achieved despite winds being generally unfavorable, i.e., still blowing predominantly with a northward component (Fig. 3). These adverse prevailing conditions can be overcome by deferring migration until occasions when the wind has a larger southward component than average. We found that this was indeed what happened: Mass migration events tended to take place when winds were blowing toward the west, compared to more northward downwind directions on nonmass migration nights (large: 275° vs. 320°, medium: 293° vs. 311°; Fig. 4 and *SI Appendix*, Table S10). So, again, there were distinct indications of wind selectivity, but in this case, making the best of suboptimal wind-transport availability and leading to displacement to the southwest. Similar selection of favorable southward-transporting winds during fall has previously been reported in the United Kingdom (2, 5, 29, 32) and North America (58, 59) and in an earlier radar study of *H. armigera* migration in northern China (38, 40).

In addition to selecting seasonally beneficial wind directions, the larger migrants also influenced their track vector by taking up heading directions that were also seemingly adaptive. During mass migrations, common orientation (defined as a unimodal distribution of individual insect headings; see *Methods*) was frequent: 90 to 100% of mass migrations in each size/season combination showed a significant degree of common orientation (*SI Appendix*, Table S1). This indicates that the comigrating insects aligned their self-powered vectors along a common bearing in response to the same environmental cues. There was a seasonal pattern, apparently adaptive, to the mean nightly heading directions, which were toward the north during spring (large: 7°, medium: 341°) and summer (large: 33°, medium: 342°) but toward the southwest or west during fall (large: 218°, medium: 275°; Fig. 3 and *SI Appendix*, Table S10). On mass-migration nights, the seasonal headings were relatively close to both the seasonal track directions and the seasonal downwind directions (compare Figs. 3 and 4). Thus, much of the insect’s airspeed is added to the wind speed, resulting in ground speeds that, for the large insects, were on average 3.7 m s^−1^ faster than the transporting winds (compare Fig. 2*B* and *SI Appendix*, Fig. S4*A*), with consequently a substantial increase in average migration distance. Orientation of the headings relatively close to the downstream direction is highly adaptive in terms of migration speed and efficiency and is a widespread phenomenon in windborne insect migration around the world (25, 60).

Thus, in summary, studies from three continents across the Northern Hemisphere all reveal that larger nocturnal insect migrants tend to fly selectively on the most optimally directed winds during spring and fall, and take up beneficial flight headings during windborne transport. This behavior is evidently adaptive as it helps migrants to take advantage of seasonal resources while reducing the risk of becoming stranded at high latitudes as winter approaches, i.e., to avoid the “Pied Piper trap” (3). Summer movements did show some differences between geographical regions, however. Over the ECP, these were largely an extension of spring movements, with generally northward displacements of both large and medium insects (Fig. 3) while in the United Kingdom, mid-summer movements of larger insects show rather random directions (5, 61). This difference can be attributed to the continuing persistence of southerly winds over the ECP through summer, a consequence of the region’s monsoon climate (62).

### Net North–South Biomass Transfer.

Across the 3 y of the study, the biomass transfer of larger nocturnal insects above the radar site in the central ECP was predominantly northward during the spring–summer migrations but predominantly southward in the fall (Fig. 1*D* and *SI Appendix*, Table S11). A mean of 4,667 t of biomass was transported annually to the north and 2,972 t to the south, i.e., the southward flow was 0.66× that to the north, a difference of 1,695 t (Fig. 1*D* and *SI Appendix*, Table S11). These values indicate that, on average, the annual northward expansion of migratory pest moths to North and Northeast China does not result in a seasonal increase in the size of the fall generation returning toward South China.

### Comparison of Insect “Bioflows” with Other Regions.

The only other region for which we have comparable estimates of insect bioflows is a 300-km wide swath of the southern United Kingdom (5). The mean annual total omnidirectional overflight per km of nocturnal insects above the ECP (15.5 billion insects km^−1^) was 5.15× greater than the equivalent value from the United Kingdom (3.02 billion km^−1^), and thus the accumulated values were 10.3× greater when comparing the entire width of each region (i.e., 600 km wide ECP vs. 300 km southern-UK region; *SI Appendix*, Table S12). When considering only the larger insect component, the ECP values were even greater: mean annual total overflights above the entire region were estimated to be 73.0× greater (237 billion larger nocturnal insects above the ECP vs. 3.24 billion above the United Kingdom; *SI Appendix*, Table S12). In addition to the much greater abundance, the high species richness of larger moths moving above the ECP (*SI Appendix*, Table S3) is also much greater than the relatively depauperate moth fauna migrating above the United Kingdom (2, 5). Due to the greater abundance of larger insects, the nocturnal biomass transfer above the ECP was considerably larger (16×) than over the southern United Kingdom (14,600 vs. 900 t; *SI Appendix*, Table S12). The greater abundance, diversity, and biomass of the nocturnal migrations above the ECP are likely explained by a combination of i) the latitudinal diversity gradient (63), as the ECP is ~18° further south than the United Kingdom; ii) the warm and wet continental summer monsoon climate of East Asia (62) being more conducive to rapid insect development and sustained nocturnal flight activity than the cooler maritime climate of the United Kingdom; and iii) the extensive area of intensive agricultural crop production in the ECP promoting large pest populations (48).

Another notable difference between the two regions is that while the northward migration over the ECP was more intense than the return southward migration in autumn, the opposite was the case in the United Kingdom where there was 3 to 4× increase between spring and fall generations of migratory *Autographa gamma* moths (3). The reasons for this difference are not clear but may include the extensive use of pesticides in China to control pests in the summer breeding grounds (19, 48, 64), plus the relative infrequency of favorable tailwinds for southward transport above the ECP during the fall migration. The consequence of these patterns is that there is a seasonal to-and-fro exchange of nutrients, biomass, energy, genes, parasites, and pathogens between the northern and southern parts of the migration arena. The northward movement also has a massive ecological impact, as numerous progeny are produced, consuming resources and being preyed upon during the summer, even though only a small proportion survive to return south in the fall.

The values above are only for night-time migration. In the UK study (5), daytime bioflows were responsible for 70% of the 24-h total. There are no measures of high-altitude daytime insect migration intensity above China or other nearby regions, but if the UK ratio holds true above the ECP, then total annual biomass transfers across the 24-h cycle may be as high as ~49,000 (47,000 to 50,000) t. These movements would be responsible for the redistribution of nutrients and energy on a massive scale, as this biomass would contain ~1,502 (1,458 to 1,546) t of nitrogen, 150 (146 to 155) t of phosphorus, and 88 (86 to 91) TJ of energy (*Methods*). The mix of species flying by day is likely to differ considerably from that of the nocturnal migrants in our samples, and the warmer conditions found in lower-latitude continental climates might make night-time flight more favored than in higher-temperate latitudes. Measurements of daytime flight activity comparable to the night-time data presented here are needed to resolve these questions.

## Conclusions

Our results, based primarily on observations from an automated, vertical-beam entomological radar, provide the first quantitative estimates of the intensity of nocturnal insect migration and the seasonal transfer of insect biomass over the intensive agricultural lands of the ECP. We show that southward return movements in fall were smaller than northward movements over the spring and summer migration periods combined, that the migrants fly predominantly on nights when the wind is blowing in the seasonally favorable direction, and that they head approximately downwind and thus add an airspeed contribution to their windborne transport toward their seasonal destination. These seasonal population movements are much more intense than similar insect migrations over the southern United Kingdom, the only other region with comparable quantitative data. However, to fully assess the implications of these northward and southward transfers for sustainable cropping, the spread of vector-borne diseases, provision of beneficial services, and general ecosystem function in this globally important agricultural region, it will be necessary to conduct a similar investigation of daytime migratory flights. With more specific information (e.g., about the migrations of individual species, rather than the broad taxon-groupings employed here), and more rapid dissemination of analyzed observations, incorporation of migration data into crop-protection forecasting and management systems may become practicable, with likely benefits for both farmers and the environment. Given increasing concerns for the health of beneficial insect populations worldwide, and likely changes in patterns of pest distribution due to climate change, natural invasions, and quarantine failures, continuing and expanded study of insect migration in this globally important agricultural region is clearly indicated.

## Methods

### Radar Observations of Larger Insect Migrants.

Long-term monitoring of larger insects flying at high altitudes was carried out nightly [19:00 to 05:00 h local time (UTC+8)] from April to October, during 2015, 2016, and 2017, with an IMR at No. 0 Entomological Radar Field Scientific Observation and Research Station of Henan Province, within the Experimental Demonstration Base for Modern Agricultural Science and Technology, Henan Academy of Agricultural Sciences (HAAS) (35.02°N, 113.69°E), located in Yuanyang county, near Zhengzhou, the capital city of Henan province (Fig. 1*A*). IMR design, operation and data analysis are described in detail elsewhere (24, 25). The IMR variant operated by HAAS is a 3.2 cm wavelength (X-band) pulsed system, with a 1.2-m diameter circular parabolic dish antenna producing a vertical-pointing, linear-polarized beam (42). The antenna feed is rotated at 5 Hz about an axis offset 0.2° from the beam center, resulting in a narrow angle conical scan combined with rotating polarization. The echo signal is digitized at 80 MHz, corresponding to height intervals of 1.875 m. Signals were recorded from insect targets passing through the beam at altitudes ranging from about 100 to 1,800 m above the radar. Recordings were made for 2.3 min every 15 min and stored as digital files. The height, track direction, displacement speed, insect body alignment, and mass of each target were calculated automatically using the time domain algorithm of Harman and Drake (65) and the mass-estimation equation of Chapman et al. (66).

We restricted our dataset to “larger” insect targets with estimated live-animal masses between 10 and 500 mg, because ambiguities in the variation of the radar cross-section with polarization angle may occur in insects weighing above ~600 mg (67), and IMR detection of small insects becomes progressively less effective at body masses below ~10 mg. This was not considered a serious limitation because very large (>500 mg) insects comprised only 1.3% of the total catch in a searchlight trap deployed near the radar site (see below), and small-insect (<10 mg) abundance was quantified with a different technique (aerial-net sampling, see below). The radar-detected insects were then divided into two categories, i.e., “medium” insects (masses 10 to 70 mg) and “large” insects (70 to 500 mg), as the migratory flight behavior can differ between the two size groups (5, 30). We also restricted our dataset to targets with displacement speed ≤17 m s^−1^, as wind speeds were almost always <13 m s^−1^ (*SI Appendix*, Fig. S4) and estimated ground speeds much higher than 17 m s^−1^ are likely to be the result of defective or misinterpreted echo signals.

To analyze seasonal migration patterns, we separated out data for peak migration occasions using the method of Hu et al. (5). First, we defined a “migration occasion” as when at least 1 insect per night was detected by our radar in the medium (10 to 70 mg) or large (70 to 500 mg) insect size category. Then, we summed all the individual targets in each season/size category combination over the 3-y dataset. The occasions which accounted for 75% of these cumulative totals were designated “mass migration” events while the remainder were termed “nonmass migration” occasions.

### Searchlight Trapping.

As an aid to determining the principal nocturnal species migrating overhead, we deployed a searchlight trap at Yuanyang (YY), about 150 m north of the radar; like the radar, it operated from April to October. This trap comprised a 1,000 W metal halide lamp and parabolic reflector (model GT70, Shanghai Yayuan Light Electronic Company, Shanghai), which projects a light beam vertically up to more than 500 m, and a large metal funnel and net for collecting insects (37). Nightly catches of insects were identified and counted every day, and insects larger than 5 mg were sampled and their masses were measured with a precision balance while still freshly dead (i.e., before any significant dehydration). Most larger insects in the searchlight catches belonged to one of three groups: moths (various families of Lepidoptera, but principally Noctuidae and Crambidae), chafer beetles (Coleoptera; Scarabaeidae), and crickets (Orthoptera; Gryllidae) (*SI Appendix*, Fig. S2). Of these, only the first group have been consistently established to be regular high-flying, windborne migrants in China, as evidenced by frequent capture of migratory moths on Beihuang Island in the center of the Bohai Strait (Northeast China) many kilometers from land (10, 47, 68). By contrast, chafers and crickets are typically absent or very rare in aerial samples and in the Beihuang Island trap catches. We therefore presumed that the chafers and crickets attracted to the lights were local insects and that Lepidoptera were the primary source of the radar echoes. We believe that searchlight trapping provides an effective method for determining the likely identity of the most abundant constituents of the radar-detected aerial fauna, as numerous studies have demonstrated the link between catches in the trap of particular species and concurrent aerial migration of the same species (10, 37–40, 46, 47, 68). As an additional check to confirm that the searchlight trap provided a representative sample of the migrant insect fauna detected by the radar, we carried out log–log regression analyses on nightly catches of the migrant fauna in the searchlight trap against nightly migration intensity of larger insects detected by the IMR, both for the entire 3-y period and for each year independently. In all cases, there were highly significant correlations (Linear regressions; whole period: *r ^2^* = 0.292, *F*_1,453_ = 188.1, *P* < 0.0001; 2015: *r ^2^* = 0.252, *F*_1,157_ = 54.3, *P* < 0.0001; 2016: *r^ 2^* = 0.377, *F*_1,134_ = 82.6, *P* < 0.0001; 2017: *r ^2^* = 0.343, *F*_1,158_ = 84.0, *P* < 0.0001; *SI Appendix*, Fig. S7).

To assess the total insect bioflow across the ECP, searchlight trapping was carried out at the four additional sites spanning the width of the plain: Mengzhou (34.91°N,112.83°E); Jiangyan (32.57°N, 120.21°N); Ninjing (37.62°N, 116.74°N); and Laizhou (37.18°N, 119.89°N) (Fig. 1*A*). Data for these sites comprised only counts of three numerous pest moth species (*A. ipsilon*, *H. armigera*, *M. separata*) and (at MZ and LZ) counts of all moth species (*SI Appendix*, Fig. S1). These traps operated from 1 May to 31 August of 2015 to 2017.

### Aerial Netting.

Aerial sampling of insects flying at ~200 m above ground was carried out between 15 August and 25 September 2009 at Jiangpu, near Nanjing, the capital city of Jiangsu province, and during 22 June to 1 July 2017 in Yuanyang county accompanying the radar observations (Fig. 1*A*). Samples were taken at night (19:00–05:00 local time) with a drogue net of 0.64 m^2^ entrance aperture suspended from a tethered helium-filled 12 m^3^ kytoon (22). Catches were sorted and later identified to genus or species.

### Weather Data.

Meteorological data, including wind direction, the component of the horizontal wind velocity toward the east, and the component toward north (*u*-wind and *v*-wind components, respectively), and air temperature, were obtained from the European Centre for Medium-Range Weather Forecasts (ECMWF) reanalysis (https://cds.climate.copernicus.eu/). Hourly weather data above the radar location during 2015 to 2017 were downloaded for eight altitudes from 975 to 800 hPa (for pressure levels every 25 hPa), which covered the vertical profiles of insect migration above the radar. Average values of these weather factors for each night (19:00–05:00 h) were matched with mass migration or nonmass migration occasions. *t* tests were used to compare the difference between environmental factors for nights of these two classes.

### Calculation of High-Altitude Migration Intensity and Biomass Transfer for Larger Insects.

The aerial density value (per 10^7^ m^3^) for individual medium and large insects detected by the IMR was calculated by an accumulation method based on the radar beamwidth and the displacement speed for each individual target (25). The aerial densities were then converted into a daily migration flow through a 1-km-wide vertical “window” from 100 m to 1,800 m above ground, running west to east above the radar (5), and this daily flow was summed for each season and each year for each of the two insect mass categories. The daily flow above the radar was then extrapolated to estimate the numbers crossing a line running west to east (i.e., perpendicular to the main north–south migration direction) along the ~600 km wide swath of the ECP. The support for this simple scaling procedure was provided by an analysis of catch sizes in the five searchlight traps spread across the plain (Fig. 1*A*). Differences among the three pest species for which data were available for all sites were examined with an ANOVA (with log-transformed catches as the dependent variable, and year, species, and site as factors).

### Calculation of High-Altitude Migration Intensity and Biomass Transfer of Small Insects.

As small insects (body masses <10 mg) are not sampled effectively by IMRs, the aerial netting data from two ECP sites were used to estimate aerial densities of both the small migrants and the larger species (see above). The ratio of larger insects to the much more numerous small insects in the catches was calculated for each site (*SI Appendix*, Table S6). The mean of these two ratios was used to estimate the number of small insects from IMR observations of the number of larger insects, while the range provides an indication of the ratio’s uncertainty. Previous studies indicated that the mean body mass of the small insects was 0.77 mg (5), so we used this value to calculate the total biomass transfer of the small-insect component of the airborne insect population.

### Calculation of Nutrient and Energy Content.

We calculated the total amounts of nitrogen (N) and phosphorus (P) in the seasonal and annual insect movements in the following way. Based on the results of Finke (69) for house crickets *Acheta domesticus*, we assumed the migrating insects were composed of 69.2% water and 30.8% dry matter. Insects are typically composed of 10% N and 1% P by dry weight (69), and thus, we were able to estimate the total N and P content of the nocturnal aerial movements. According to Finke (69), each kg of the dry mass of *A. domesticus* has an energy content of 1,402 kcal, which is equivalent to 5.87 × 10^6^ J kg^−1^; we applied this value to the estimated biomass transfer to estimate the total energy content of the seasonal insect migrations over the ECP.

### Assessment of Seasonal Migration Track, Flight Heading, and Downwind Directions.

For each mass-migration event of the larger insects, three circular statistics (70) were calculated for each of the angular distributions of directions: i) the mean direction; ii) the mean resultant vector length “*r*” (a measure of the clustering of the tracks or headings, ranging from 0 to 1, with higher values indicating greater deviations from a uniform distribution); and iii) the probability that the distribution of directions differed from a uniform distribution according to the Rayleigh test (for which a significance level of *P* < 0.05 was adopted). Mass-migration events with a significant unimodal distribution of tracks and/or headings were then used to calculate the overall mean directions of these quantities for each size category and each season. Similar processes were carried out with seasonal patterns of downwind directions, but in addition, winds on mass-migration nights were compared with winds on non-mass-migration nights. The radar measures insect body alignment rather than heading direction per se, so there is initially a 180° ambiguity regarding the true heading. However, high-altitude migrants in northern temperate regions nearly always orient relatively close to the downwind direction (2, 25, 37–40), so of the two possible axial directions, the heading value that was closest to the track direction was selected as the true heading direction.

## Supplementary Material

Appendix 01 (PDF)

## Data Availability

Excel spreadsheets with estimates of aerial densities and migration rates for 2015 to 2017, derived from IMR data, have been deposited in the Dryad repository, https://doi.org/10.5061/dryad.8sf7m0cxh (71).
