# Supplementary material for: Massive seasonal high-altitude migrations of nocturnal insects above the agricultural plains of East China
Source: Proc Natl Acad Sci U S A. 2024 Apr 22;121(18):e2317646121. doi: 10.1073/pnas.2317646121 (PMC11067063; doi:10.1073/pnas.2317646121)
Supplement: Supplementary file 1 — Appendix 01 (PDF) [file pnas.2317646121.sapp.pdf]

Supporting information

Massive seasonal high-altitude migrations of nocturnal insects above the agricultural plains of East China

Jianrong Huang, Hongqiang Feng, V. Alistair Drake, Don R. Reynolds, Boya Gao, Fajun Sun, Guoyan Zhang, Junsheng Zhu, Yuebo Gao, Baoping Zhai, Guoping Li, Caihong Tian, Bo Huang, Gao Hu, Jason W. Chapman

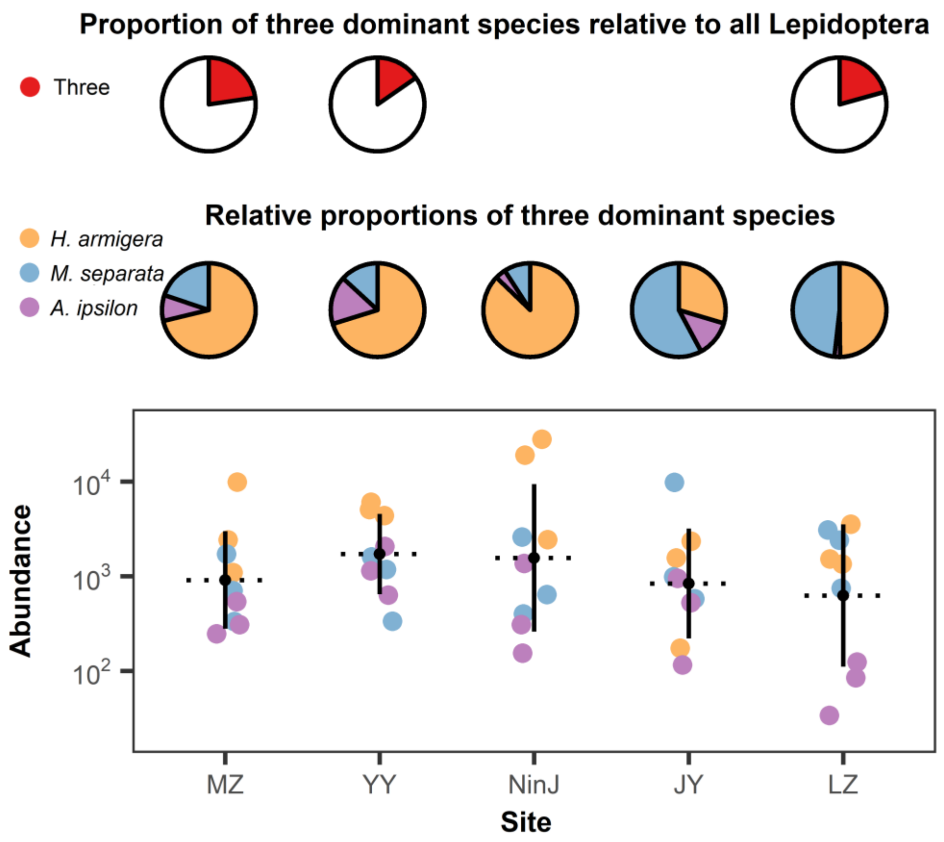

Fig. S1. Annual total searchlight-trap catches of three pest noctuid moth species, black cutworm *Agrotis ipsilon* (purple), cotton bollworm *Helicoverpa armigera* (orange), and oriental armyworm *Mythimna separata* (blue), from five monitoring sites in the East China Plain from 1 May to 31 August in 2015–2017. Horizontal dotted lines and the vertical error bar in the lower plot represent the mean values ± 1 S.D. Searchlight traps were operated at: Mengzhou City (MZ), Henan; Yuanyang County (YY), Henan; Ningjin County (NinJ), Shandong; Jiangyan District (JY), Taizhou City, Jiangsu; and Laizhou City (LZ), Shandong.

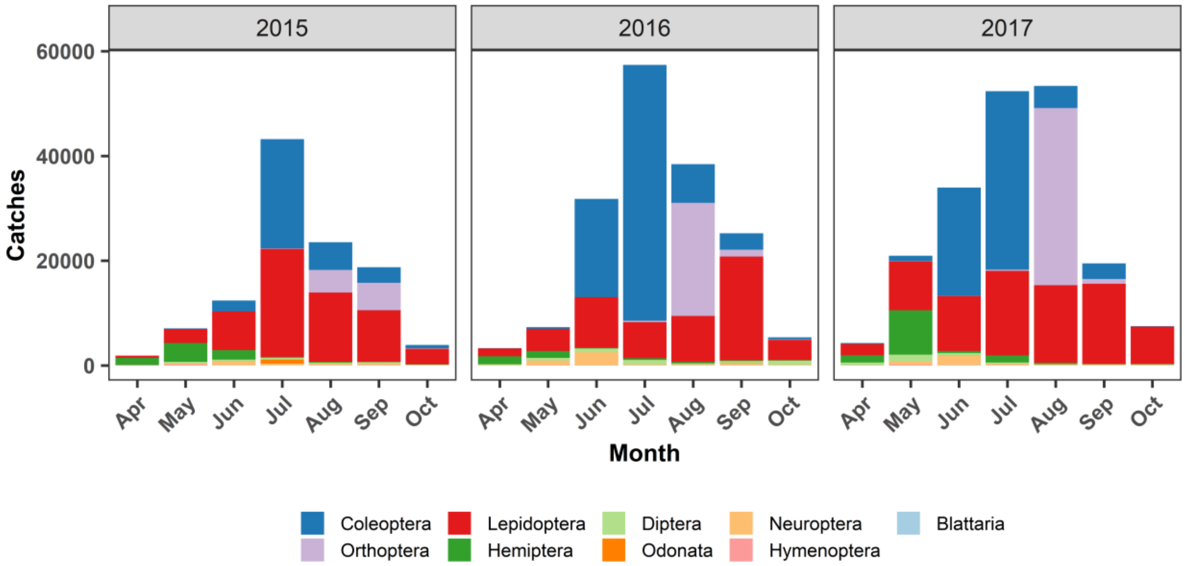

Fig. S2. The annual numbers of insects of each order caught in a searchlight trap at Yuanyang County, Henan (see Fig. 1*A*), in 2015–2017. A total of 176 species, comprising 435,532 individuals, were trapped from 1 April to 31 October, with most being caught between June and September. Lepidoptera, Coleoptera and Orthoptera were the most abundant orders. Large insects (70–500 mg) were represented by 79 species of Lepidoptera (including 53 Noctuidae), and another 17 species from 8 other insect orders. Medium insects (10–70 mg) were represented by 43 species of Lepidoptera (including 17 Crambidae and 10 Noctuidae), 15 species of Coleoptera, and 13 species belonging to five other orders. All these species were large enough to be readily detected by the Insect Monitoring Radar. Also see Tables S2 and S3 for a complete taxonomic breakdown of the various insect orders sampled in the searchlight traps, and a complete list of the Lepidoptera species in the samples.

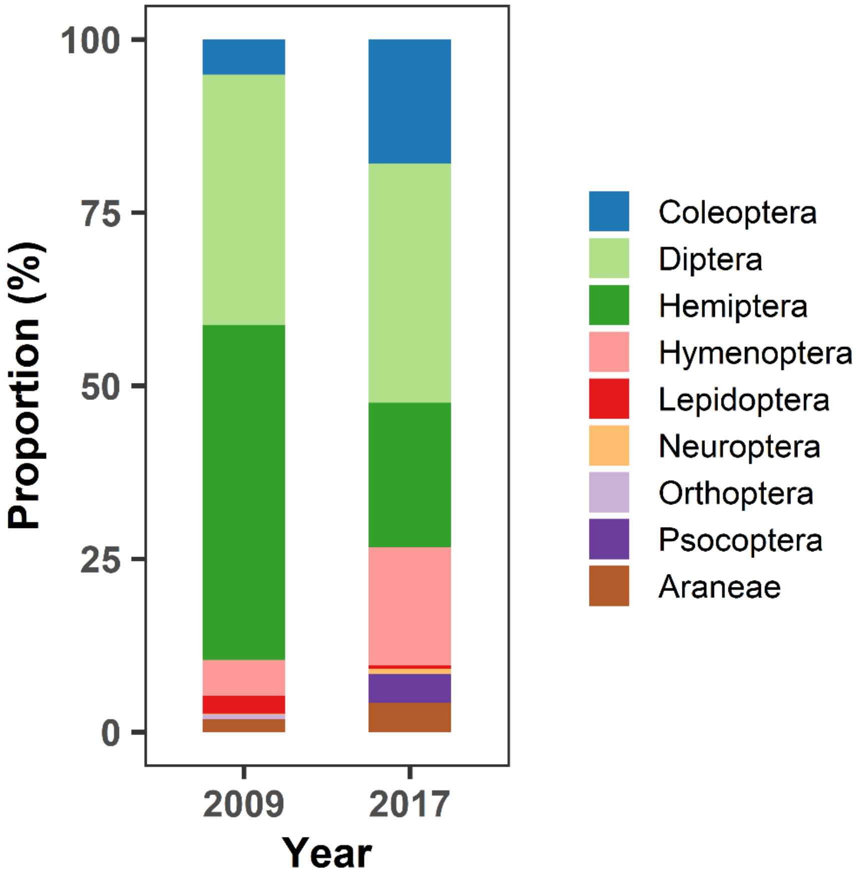

Fig. S3. The proportions of insects from each order in night-time aerial netting samples taken at Jiangpu (32.01°N, 118.62°E), Jiangsu province in 2009 and at Yuanyang County, Henan province in 2017. The netting samplings were dominated by Hemiptera, Diptera and Hymenoptera, which cumulatively accounted for 84.5% and 55.4% of the totals in 2009 and 2017, respectively. See also Table S7 for a taxonomic breakdown of abundances in the aerial samples.

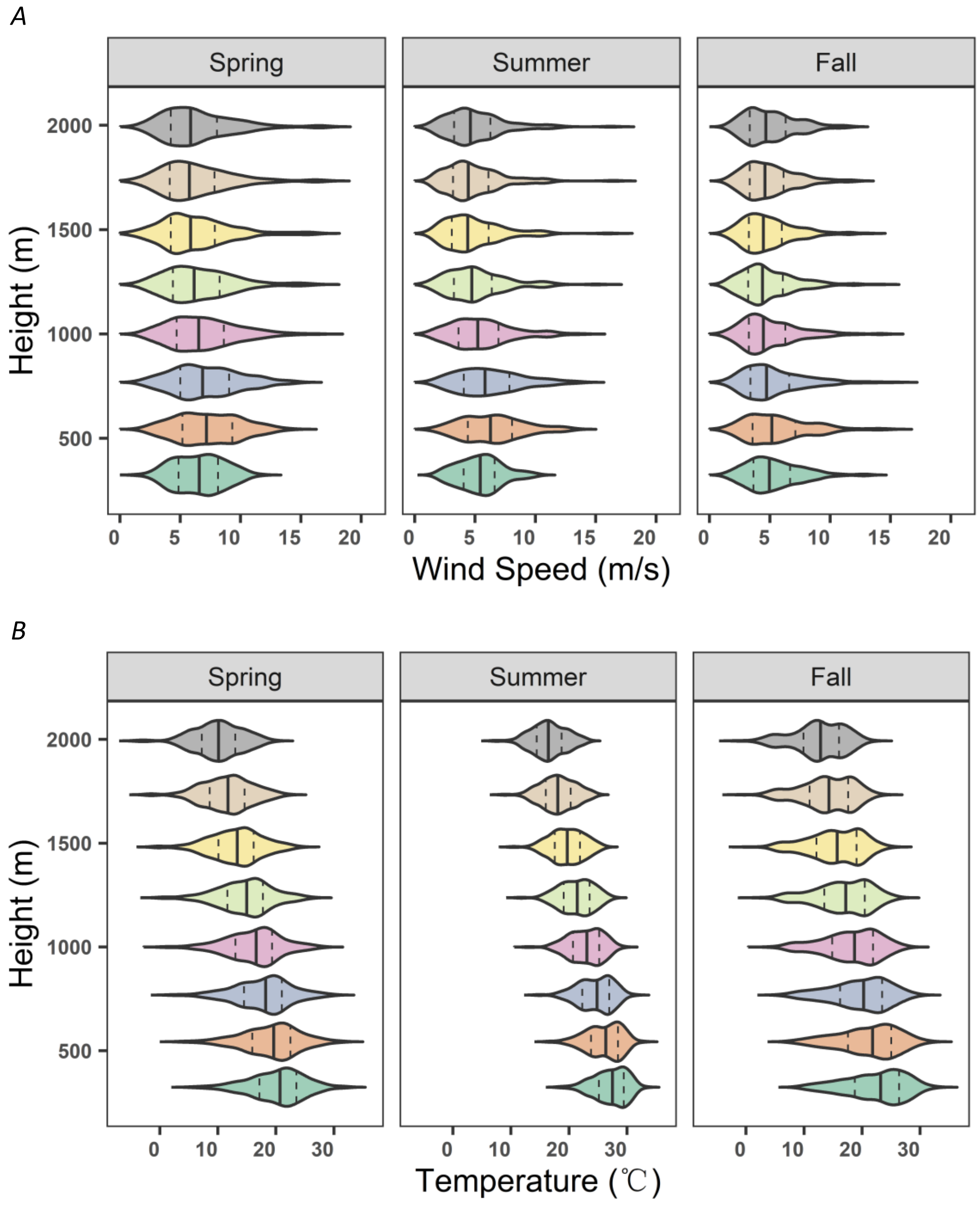

Fig. S4. Windspeeds (*A*) and air temperatures (*B*) at a range of altitudes above ground level during all migration events. In the violin plots, solid black lines represent the median, dashed black lines represent the inter-quartile range (IQR). Data from ECMWF reanalyzes for every night in the 2015-2017 study period, averaged over 1 h intervals; nominal heights correspond to 975 to 800 hPa pressure levels at 25 hPa intervals.

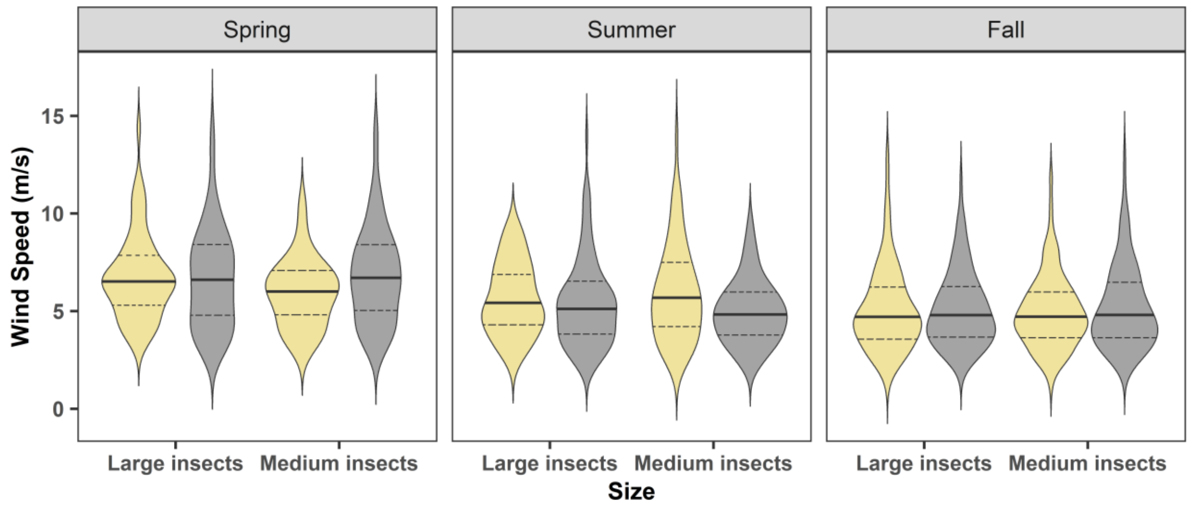

Fig. S5. Wind speeds during "mass migrations" (yellow) and "non-mass migrations" (gray) between 100–1800 m above ground. In the violin plots, solid black lines represent the median, dashed black lines represent the inter-quartile range (IQR). The mean wind speeds during mass migrations were not significantly different to wind speeds during non-mass migrations in all three seasons for large insects (mean speed ± SD; Spring: mass migrations, 6.5 ± 2.2 m s^{-1} vs non-mass, 6.9 ± 1.9 m s^{-1}, t = 0.31, P = 0.76; Summer: mass migrations, 5.3 ± 2.2 m s^{-1} vs non-mass, 5.4 ± 2.6 m s^{-1}, t = 0.58, P = 0.56; Fall: mass migrations, 5.2 ± 1.8 m s^{-1} vs non-mass, 5.2 ± 2.2 m s^{-1}, t = -0.04, P = 0.96). They were also no different for medium insects during mass vs non-mass migration in fall (mass migrations, 5.1 ± 2.5 m s^{-1} vs non-mass, 5.3 ± 1.6 m s^{-1}, t = -0.84, P = 0.40), but there were small but significant differences in spring (mass migrations, 6.0 ± 1.9 m s^{-1} vs non-mass, 7.0 ± 2.1 m s^{-1}, t = -2.12, P = 0.04) and summer (mass migrations, 5.9 ± 1.7 m s^{-1} vs non-mass, 5.0 ± 1.6 m s^{-1}, t = 2.61, P = 0.01). Wind data from every night in the study period (2015-2017) from 800 to 975 hPa in 25 hPa intervals and averaged over 1-hour intervals. Wind dataset as in Fig.S4, but partitioned according to category of migration event, first for large insects and then for medium insects.

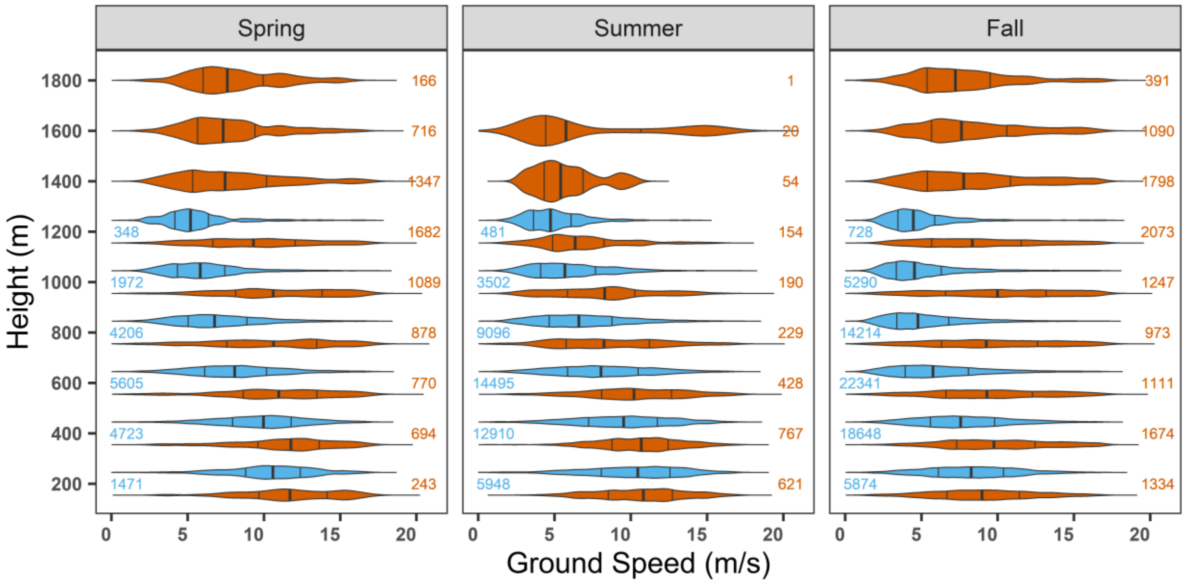

Fig. S6. Vertical profiles of the ground speed recorded by the IMR during "mass migrations" of large (brown) and medium (blue) insects in each season and in each 200 m-deep height intervals. In the violin plots, wide vertical lines represent the median, thin vertical lines represent the inter-quartile range (IQR). Long upper tails, especially evident in large insects, are thought to represent defective or misinterpreted echo signals, and targets with ground speeds >17 m s^{-1} have been eliminated from the analyses.

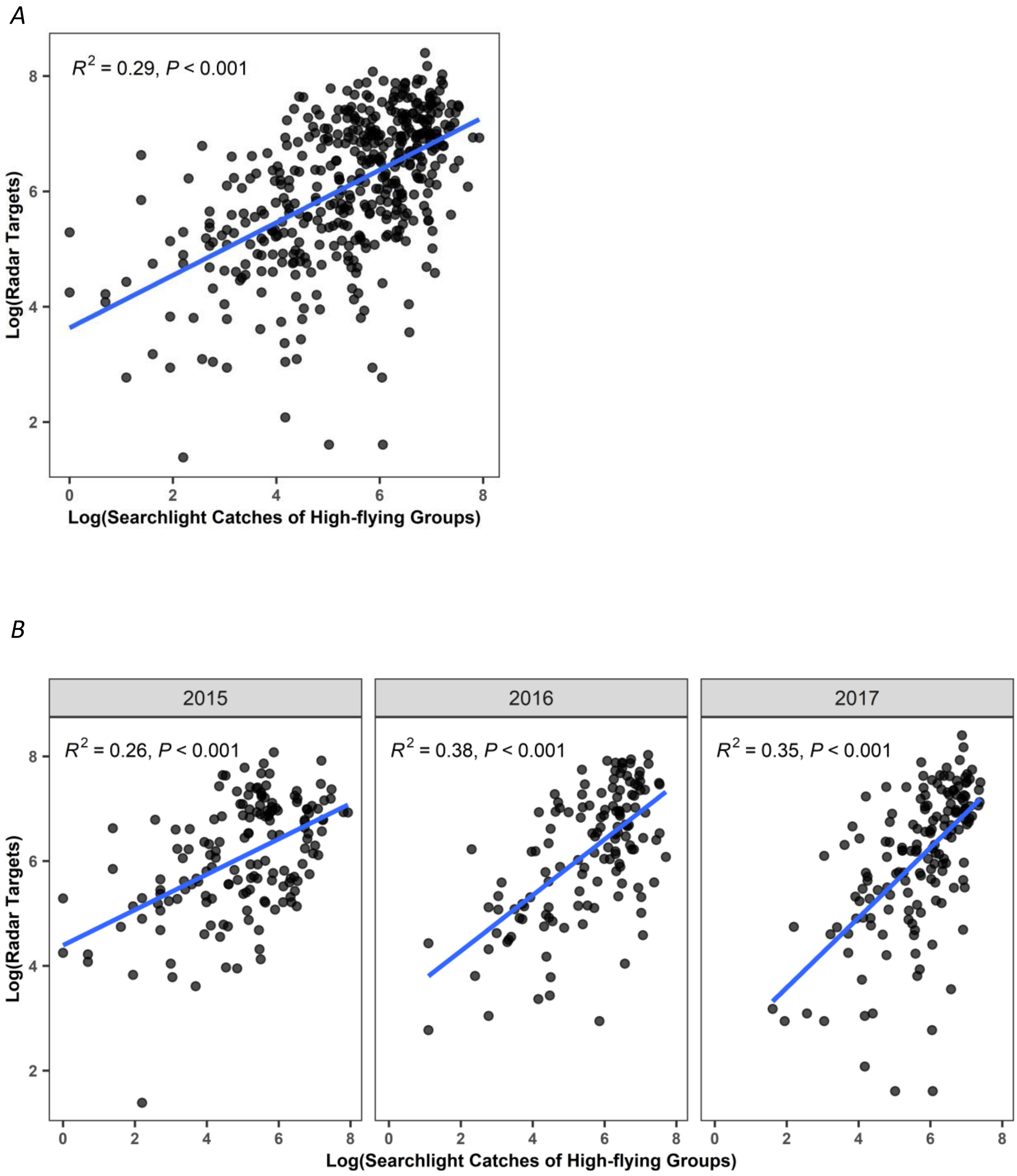

Fig. S7. Log-log linear regression analyses of nightly catches of migrant fauna in the searchlight trap at Yuanyang, and the migration intensity of larger insects detected by the accompanying IMR. (*A*) The entire three-year study period; (*B*) each of the three years analyzed independently.

Table S1. Total number of migration occasions, number of "mass migrations", and numbers of individual larger insects detected by the IMR. The proportion of the mass migrations which exhibited a significant degree of common orientation (see *Methods*) is also presented. Mass migrations are defined as the subset of occasions which cumulatively account for 75% of the total number of individuals in each size category across the year.

Insect category	Season	Month	Number of migration occasions		Number of insects		Number of mass migrations with common orientation
			All migrations	Mass migrations (%)	All migrations	Mass migrations (%)	
**Large** (70–500 mg)	Spring	Apr	64	10 (15.6%)	4,134	2,055 (49.7%)	10 (100%)
		May	84	51 (60.7%)	14,705	13,244 (90.1%)	50 (98%)
		Subtotal	148	61 (41.2%)	18,839	15,299 (81.2%)	60 (98.4%)
	Summer	Jun	58	8 (13.8%)	3,313	1,286 (38.8%)	7 (87.5%)
		Jul	73	19 (26%)	5,856	3,561 (60.8%)	19 (100%)
		Subtotal	131	27 (20.6%)	9,169	4,847 (52.9%)	26 (96.3%)
	Fall	Aug	75	32 (42.7%)	10,977	8,800 (80.2%)	30 (93.8%)
		Sep	73	48 (65.8%)	14,007	12,457 (88.9%)	44 (91.7%)
		Oct	67	10 (14.9%)	5,147	2,117 (41.1%)	10 (100%)
		Subtotal	215	90 (41.9%)	30,131	23,374 (77.6%)	84 (93.3%)
	**Grand total**	**All**	**494**	**178 (36%)**	**58,139**	**43,520 (74.9%)**	**170 (95.5%)**
**Medium** (10–70 mg)	Spring	Apr	65	0	7717	0	-
		May	84	25 (29.8%)	50,930	34,638 (68%)	25 (100%)
		Subtotal	149	25 (16.8%)	58,647	34,638 (59.1%)	25 (100%)
	Summer	Jun	59	31 (52.5%)	53,735	42,245 (78.6%)	31 (100%)
		Jul	73	34 (46.6%)	57,531	43,578 (75.7%)	34 (100%)
		Subtotal	132	65 (49.2%)	111,266	85,823 (77.1%)	65 (100%)
	Fall	Aug	75	42 (56%)	71,060	58,287 (82%)	41 (97.6%)
		Sep	73	46 (63%)	78,379	68,076 (86.9%)	46 (100%)
		Oct	67	0	10,742	0	-
		Subtotal	215	88 (40.9%)	160,181	126,363 (78.9%)	87 (98.9%)
	**Grand total**	**All**	**496**	**178 (35.9%)**	**330,094**	**246,824 (74.8%)**	**177 (99.4%)**

Table S2. Total abundance and relative proportion of all insect taxa, and those considered to be high-flying migrants, in the searchlight trap catches at the radar site in Yuanyang County, Henan province of China from 1 April to 31 October in 2015–2017.

Size	Order	Family	Total (%)	High-flying groups (%)
Large (70~500 mg)	Blattodea	Blattidae	414 (0.10%)	Y (0.18%)
	Coleoptera	Carabidae	16,009 (3.68%)	Y (7.04%)
		Melolonthidae	136,341 (31.31%)	N
	Diptera	Asilidae, Tipulidae	4 (0%)	Y (<0.01%)
	Dermaptera	Labiduridae,	14 (0%)	Y (0.01%)
	Hemiptera	Cicadidae, Miridae, Pentatomidae	93 (0.02%)	Y (0.04%)
	Lepidoptera	Noctuidae, etc.	64,642 (14.85%)	Y (28.43%)
	Odonata	Aeshnidae	863 (0.2%)	Y (0.38%)
	Orthoptera	Acrididae	1,230 (0.28%)	Y (0.54%)
		Grylloidea	64,821 (14.89%)	N
	**Sub-Total**		**284,431 (65.31%)**	**83,383 (36.63%)**
Medium (10~70 mg)	Coleoptera	Melolonthidae	7,016 (1.61%)	N
		Other Coleoptera (e.g., Coccinellidae, etc.)	14,661 (3.37%)	Y (6.45%)
	Diptera	Muscidae, Tipulidae, Syrphidae	8,096 (1.86%)	Y (3.56%)
	Hemiptera	Pentatomidae, etc.	3,038 (0.7%)	Y (1.34%)
	Hymenoptera	Apidae	519 (0.12%)	Y (0.23%)
	Lepidoptera	Noctuidae, Crambidae, Pyralidae, etc.	108,647 (24.95%)	Y (47.79%)
	Neuroptera	Chrysopidae	9,016 (2.07%)	Y (3.97%)
	Odonata	Libellulidae	108 (0.02%)	Y (0.05%)
	**Sub-Total**		**151,101 (34.69%)**	**143,971 (63.37%)**
**Grand Total**			**435,532**	**227,354**
Total Lepidoptera			173,289 (39.79%)	173,289 (76.22%)

Note: “Y” indicates this family are regular high–flying groups and “N” represents groups not suspected to be regular high-flying migrants.

Table S3. Total abundance and relative proportion of the 122 species of larger Lepidoptera (>10 mg) caught in the searchlight trap at the radar site in Yuanyang County, Henan, from 1 April to 31 October 2015–2017. The 10 most abundant species are in bold blue font; 80 pests of crops and ornamental plants are shaded grey.

Family	Species	Total (%)	Family	Species	Total (%)
		**Large (70-500 mg)**			
**Cossidae**	*Olcocerus vicarious*	33 (0.02)	**Noctuidae**	*Diarsia canescens*	768 (0.44)
**Ctenuchidae**	*Amata emma*	17 (0.01)		*Eligma narcissus*	54 (0.03)
**Erebidae**	*Amsacta lactinea*	14 (0.01)		*Eublemma cochylioides*	7 (<0.01)
	Arctor nisalba	59 (0.03)		*Eutelia geyeri*	16 (0.01)
	Calliteara pudibunda	2 (<0.01)		*Euxoa oberthuri*	109 (0.06)
	Euproctis chrysorrhoea	36 (0.02)		***Helicoverpa armigera***	**21,609 (12.47)**
	Euproctis flava	1 (<0.01)		*Helicoverpa assulta*	16 (0.01)
	Spilarctia subcarnea	81 (0.05)		*Heliothis dipsacea*	8 (<0.01)
	Spilosoma menthastri	96 (0.06)		*Lagoptera juno*	34 (0.02)
	Spilosoma nivea	6 (<0.01)		***Leucania loreyi***	**7,857 (4.53)**
	Spilosoma niveus	38 (0.02)		*Leucania venalba*	10 (0.01)
	Stigmatophora flava	8 (<0.01)		*Macdunnoughia crassisigna*	1,535 (0.89)
	Stilprotia salicis	113 (0.07)		*Mamestra brassicae*	843 (0.49)
**Geometridae**	*Ascotis selenaria*	684 (0.39)		*Mocis ancilla*	1 (<0.01)
	Calospilos suspecta	2,583 (1.49)		*Mythimna rufipennis*	4 (<0.01)
	Lomographa margarita	2 (<0.01)		***Mythimna separata***	**3,432 (1.98)**
	Ourapteryx nivea	1 (<0.01)		*Mythimna turca*	25 (0.01)
	Percnia giraffata	1 (<0.01)		*Mythimna velutina*	10 (0.01)
**Lasiocampidae**	*Gastropacha populifolia*	58 (0.03)		*Ophiusa tirhaca*	2 (<0.01)
	Gastropacha quercifolia	26 (0.02)		*Oraesia excavate*	30 (0.02)
	Adris tyrannus	1 (<0.01)		*Parallelia arctotaenia*	129 (0.07)
**Limacodidae**	*Cnidocampa flavescens*	130 (0.08)		*Parallelia stuposa*	147 (0.08)
**Noctuidae**	*Abrostola triplasia*	106 (0.06)		*Peridroma saucia*	3,210 (1.85)
	Acronicta major	98 (0.06)		*Perigrapha circumducta*	1 (<0.01)
	Acronicta rumicis	1,357 (0.78)		*Plusia orichalcea*	2 (<0.01)
	Agrotis segetum	1,968 (1.14)		*Polia illoba*	653 (0.38)
	Agrotis tokionis	12 (0.01)		*Protoschinia scutosa*	105 (0.06)
	Agrotis ipsilon	**4,179 (2.41)**		*Pyrrhia umbra*	17 (0.01)
	Anarta trifolii	3,036 (1.75)		*Sesamia inferens*	814 (0.47)
	Anomis mesogona	26 (0.02)		*Sidemia depravata*	1,490 (0.86)
	Anua trapezium	67 (0.04)		*Spodoptera litura*	273 (0.16)
	Argyrogramma albostriata	1,738 (1.00)		*Trachea atriplicis*	470 (0.27)
	Autographa nigrisigna	178 (0.1)		*Trigonodes hyppasia*	2 (<0.01)
	Axylia putris	377 (0.22)		*Xanthodes transversa*	563 (0.32)
	Brithys crini	34 (0.02)		*Xestia c-nigrum*	312 (0.18)
	Chrysaspidia festucae	29 (0.02)	**Notodontidae**	*Cerura menciana*	70 (0.04)
	Cocytodes coerulea	5 (<0.01)		*Clostera anachoreta*	200 (0.12)
	Ctenoplusia agnata	2,062 (1.19)		*Nerice davidi*	33 (0.02)
	Cucullia fraterna	12 (0.01)	**Sphingidae**	*Teretra japonica*	503 (0.29)
	Cucullia splendida	4 (<0.01)			

Medium (10-70 mg)					
**Crambidae**	*Agriphila aeneociliella*	985 (0.57)	**Geometridae**	*Napocheima robiniae*	10 (<0.01)
	Ancylolomia japonica	273 (0.16)		*Percnia longitermen*	14 (0.01)
	Botyodes diniasalis	**4,740 (2.74)**		*Semiothisa cinerearia*	1,203 (0.69)
	Chilo sacchariphagus	4 (<0.01)	**Limacodidae**	*Latoia sinica*	11 (0.01)
	Chilo suppressalis	21 (0.01)	**Noctuidae**	*Agrotis corticea*	8 (<0.01)
	Conogethes punctiferalis	**8,758 (5.05)**		*Anomis flava*	89 (0.05)
	Diaphania indica	541 (0.31)		*Anophia leucomelas*	1,344 (0.78)
	Diaphania perspectalis	106 (0.06)		***Athetis lepigone***	**36,957 (21.33)**
	Diaphania quadrimaculalis	164 (0.09)		*Earias pudicana*	2,253 (1.3)
	Haritalodes derogata	162 (0.09)		*Earias roseifera*	234 (0.14)
	Hymenia recurvalis	**8,872 (5.12)**		*Emmelia trabealis*	37 (0.02)
	Maruca testulalis	259 (0.15)		*Maliattha signifier*	376 (0.22)
	Nomophila nocteulla	321 (0.19)		*Rivula sericealis*	6 (<0.01)
	Notarcha basipunctalis	204 (0.12)		***Spodoptera exigua***	**26,331 (15.19)**
	Omphisa plagialis	196 (0.11)	**Notodontidae**	*Micromelalopha troglodyta*	2,733 (1.58)
	Ostrinia nubilalis	**5,894 (3.40)**		*Pterostoma sinicum*	38 (0.02)
	Palpita nigropunctalis	424 (0.24)	**Pieridae**	*Pieris rapae*	1(<0.01)
**Erebidae**	*Hyphantria cunea*	278 (0.16)	**Pyralidae**	*Etiella zinckenella*	2,567 (1.48)
**Gelechiidae**	*Brachmia macroscopa*	77 (0.04)		*Oncocera semirubella*	632 (0.36)
**Geometridae**	*Calothysanis comptaria*	642 (0.37)		*Orthopygia glaucinalis*	691 (0.40)
	Jinchihuo honesta	33 (0.02)	**Tortricidae**	*Pandemis heparana*	44 (0.03)
	Macaria shanghai	115 (0.07)			
				**Grand Total**	**173,289 (100)**

Table S4. Taxonomic composition of insects sampled in aerial nets about 200 m above the ECP. Samples from nights of 15 August – 25 September 2009 at Jiangpu, Jiangsu, China (32.01°N, 118.62°E).

Order	Major Families	Species	Number individuals <10 mg	Number individuals >10 mg
**Hemiptera**	Delphacidae	*Sogatella furcifera*	58	
		Laodelphax striatellus	43	
		Nilaparvata spp.	13	
		Other Delphacids	239	
	Cicadellidae	*Cicadella viridis*		7
		Other Cicadellids	148	
	Miridae	Small mirid spp.	10	
		Cytorhinus lividipennis		7
	Veliidae	*Microvelia spp.*	63	
	Aphididae	Aphid spp.	271	
	Other Families	Unidentified spp.	49	
**Diptera**	Chironomidae	Unidentified spp.	392	
	Culicidae	Unidentified spp.	27	
	Cecidomyiidae	*Rhopalomyia*	44	
	Ceratopogonidae	Unidentified spp.	15	
	Chaoboridae	Unidentified spp.	46	
	Agromyzidae	Unidentified spp.	51	
	Muscidae	*Atherigona spp.*	35	
	Other Families	Unidentified spp.	16	
**Coleoptera**	Carabidae	Unidentified spp.		9
	Coccinellidae	*Coccinella septumpunctata*		1
	Staphylinidae	Unidentified spp.	49	
	Other families	Unidentified spp.	33	
**Hymenoptera**	Ichneumonidae	Unidentified sp.		1
	Other Families	Unidentified spp.	93	
**Orthoptera**	Acrididae	*Oxya chinensis*		1
		Unidentified sp.		11
	Tridactylidae	Unidentified sp.		1
**Lepidoptera**	Crambidae	*Cnaphalocrocis medinalis*	30	
		Maruca testulalis		3
	Pyralidae	Unidentified sp.		4
	Sessidae	Unidentified sp.		1
	Pieridae	Unidentified sp.		1
	Erebidae	*Blavia scoteola*	3	
	Tineidae	Unidentified sp.	4	
**Trichoptera**		Unidentified sp.	1	
**Neuroptera**	Chrysopidae	Unidentified sp.		2
**Spiders**			33	
**TOTALS**			**1766**	**49**

Table S5. Taxonomic composition of insects sampled in aerial nets about 200 m above the ECP. Samples from the nights of 22 Jun, 23 Jun, and 27 Jun - 1 Jul 2017 at Yuanyang county, Henan (35.02°N, 113.69°E).

Order	Major Families	Species	Number individuals <10 mg	Number individuals >10 mg
**Hemiptera**	Delphacidae	Unidentified sp.	26	
	Cicadellidae	Unidentified sp.	21	
	Aphididae	Unidentified sp.	56	
	Psylloidea	Unidentified sp.	3	
	Miridae	Unidentified sp.	1	
	Lygaeidae	*Nysius ericae*	74	
	Other Families	Unidentified sp.	2	
**Diptera**	Cecidomyiidae	Unidentified sp.	8	
	Sciaridae	Unidentified sp.	16	
	Ceratopogonidae	Unidentified sp.	6	
	Chironomidae	Unidentified sp.	9	
	Phoridae	Unidentified sp.	115	
	Drosophilidae	Unidentified sp.	84	
	Chloropidae	Unidentified sp.	6	
	Culicidae	Unidentified sp.	3	
	Other Families	Unidentified sp.	56	
**Coleoptera**	Carabidae	*Notiophilus rufipes*		1
		Other Carabidae	3	
	Staphylinidae	Unidentified sp.		12
	Coccinellidae	Unidentified sp.		2
	Cryptophagidae	Unidentified sp.	47	
	Chrysomelidae	Unidentified sp.	1	
	Latridiidae	Unidentified sp.	88	
	Other Families	Unidentified sp.	3	
**Hymenoptera**		Unidentified sp.	150	
**Lepidoptera**	Geometridae	*Nycterosea obstipata*	1	
	Other Families	Unidentified sp.	3	
**Neuroptera**	Chrysopidae	*Chrysoperla sinica*		6
**Psocoptera**	Psocidae	Unidentified sp.	37	
**Araneae**			37	
**TOTALS**			**856**	**21**

Table S6. The ratio of small insects (<10 mg) to larger insects (10–500 mg) in night-time aerial netting samples taken at ~200 m above ground at two locations in the ECP.

Location	Sampling Dates	Numbers[a]		Ratio small to larger insects
		larger insects >10mg	small insects <10mg	
Jiangpu, China, 32.01°N, 118.62°E	15 August – 25 September 2009	49	1,766	36.04
Yuanyang, China, 35.02°N, 113.69°E	22 Jun, 23 Jun, 27 Jun - 1 Jul 2017	21	856	40.76
**Mean**				**38.40**

Note. [a]From Tables S4 and S5.

Table S7. Abundance of key groups of small insects in aerial netting surveys carried out above Jiangpu, Jiangsu province,and Yuanyang, Henan province in ECP.

Order	**2009 Jiangpu**		**2017 Yuanyang**		**Mean**
	**No.**	**%**	**No.**	**%**	**No. (%)**
Hemiptera	877	48.3	183	20.9	530 (39.3%)
Diptera	657	36.2	303	34.5	480 (35.6%)
Coleoptera	92	5.1	157	17.9	125 (9.3%)
Hymenoptera	94	5.2	150	17.1	122 (9.1%)
Orthoptera	13	0.7	0	0	7 (0.5%)
Lepidoptera	47	2.6	4	0.5	26 (1.9%)
Neuroptera	2	0.1	6	0.7	4 (0.3%)
Psocoptera	0	0	37	4.2	18 (1.3%)
Araneae	33	1.8	37	4.2	35 (2.6%)

Table S8. Annual cumulative omnidirectional flows of nocturnal insect migrants above the ECP

Year	Number of insects in billions (×10⁹) (km⁻¹)				Number of insects in trillions (×10¹²) per 600 km [a]			
	Large Insects >70 mg (%)	Medium Insects 10~70 mg (%)	Small insects <10 mg (%)[bc]	Total	Large Insects	Medium Insects	Small insects	Total
2015	0.019 (0.13%)	0.362 (2.41%)	14.6 (13.7~15.5) (97.46%)	15.0 (14.1~15.9)	0.011	0.217	8.8 (8.2~9.3)	9.0 (8.5~9.5)
2016	0.033 (0.21%)	0.356 (2.33%)	14.9 (14.0~15.8) (97.46%)	15.3 (14.4~16.2)	0.020	0.214	9.0 (8.4~9.5)	9.2 (8.6~9.7)
2017	0.030 (0.18%)	0.384 (2.36%)	15.9 (14.9~16.9) (97.46%)	16.3 (15.3~17.3)	0.018	0.230	9.5 (9.0~10.1)	9.8 (9.2~10.4)
**Mean**	**0.027** (0.17%)	**0.367** (2.37%)	**15.1 (14.2~16.1)** (97.46%)	**15.5 (14.6~16.5)**	**0.016**	**0.220**	**9.1 (8.5~9.6)**	**9.3 (8.8~9.9)**

Note: [a]Scaled from columns 2-5 for representative 600 km width of the ECP; proportions (%) not shown as same as in columns 2-4. [b]Ranges from difference of small:larger ratio values in 2009 and 2017 aerial-trapping samples. [c]Estimated small insect proportion is the same for all rows as it is determined from the same small/larger ratio (Table S6).

Table S9. Annual cumulative omnidirectional biomass transfer of nocturnal insect migrants above the ECP

Year	Total biomass transfer (t km^{-1})			Total biomass transfer (t) per 600 km		
	Larger insects	Small insects	Total	Larger insects	Small insects	Total
2015	10.6 (48.6%)	11.3 (51.4%) (10.6-11.9)	21.9 (21.2-22.6)	6,390	6,750 (6330-7160)	13,100 (12,700-13,500)
2016	13.7 (54.3%)	11.5 (45.7%) (10.8-12.2)	25.2 (24.4-25.9)	8,200	6,890 (6460-7310)	15,000 (14,600-15,500)
2017	13.9 (53.2%)	12.2 (46.8%) (11.5-13.0)	26.1 (25.4-26.9)	8,330	7,340 (6890-7790)	15,600 (15,200-16,100)
**Mean**	**12.7 (52.2%)**	**11.7 (47.8%) (10.9-12.4)**	**24.4 (23.7-25.1)**	**7,640**	**6,990 (6560-7420)**	**14,600 (14,200-15,000)**

Note. Notes a-c of Table S8 also apply here.

Table S10. Mean directions and associated circular statistics from Rayleigh tests for downwind directions during all night-time occasions, and for track and heading directions during mass insect migrations. Data for 2015-2017; insect means are for all detected targets during mass migration nights; wind data from every night in the study period from 800 to 975 hPa in 25 hPa intervals, and averaged over 1-hour intervals throughout the night (19:00 – 05:00 h local time). These data are shown in Figs. 3 and 4. Note: wind direction is downwind direction.

		**Directional Data**	***n***	**Mean Direction (°)**	***r***	***P***
**Spring**	All	Wind (all nights)	149	27.6	0.351	<0.001
	Large Insects	Insect track	60	352.5	0.964	<0.001
		Insect heading	54	7	0.882	<0.001
		Wind (non-mass)	87	14	0.227	0.011
		Wind (mass migration)	61	10.6	0.574	<0.001
	Medium Insects	Insect track	25	351.3	0.880	<0.001
		Insect heading	25	341.4	0.669	<0.001
		Wind (non-mass)	124	11	0.251	<0.001
		Wind (mass migration)	25	10	0.787	<0.001
**Summer**	All	Wind (all nights)	132	348.2	0.472	<0.001
	Large Insects	Insect track	26	2.2	0.67	<0.001
		Insect heading	25	33.4	0.705	<0.001
		Wind (non-mass)	104	334.2	0.453	<0.001
		Wind (mass migration)	27	6.7	0.816	<0.001
	Medium Insects	Insect track	65	326.8	0.567	<0.001
		Insect heading	65	342.2	0.355	<0.001
		Wind (non-mass)	67	330.5	0.478	<0.001
		Wind (mass migration)	65	351.5	0.568	<0.001
**Fall**	All	Wind (all nights)	215	293.8	0.249	<0.001
	Large Insects	Insect track	84	205.3	0.728	<0.001
		Insect heading	75	218.2	0.618	<0.001
		Wind (non-mass)	125	320.4	0.351	<0.001
		Wind (mass migration)	90	275.1	0.342	<0.001
	Medium Insects	Insect track	88	243	0.372	<0.001
		Insect heading	87	274.6	0.421	<0.001
		Wind (non-mass)	127	311.2	0.238	0.001
		Wind (mass migration)	88	292.9	0.419	<0.001

Table S11. Net biomass transfer (metric tons) of larger nocturnal insects in the central ECP.

Year	Spring		Summer		Fall		Sum of N	Sum of S	Net flux	Ratio S/N
	N	S	N	S	N	S				
2015	1302	64	1503	538	1003	1975	3807	2577	1,230	0.68
2016	1185	12	1378	636	1836	3150	4399	3798	601	0.86
2017	2044	18	2063	634	1687	1889	5794	2540	3,253	0.44
**mean**	**1510**	**32**	**1648**	**603**	**1509**	**2338**	**4667**	**2972**	**1,695**	**0.66**

Note: N and S are northward and southward components of biomass flow.

Table S12. Comparison of abundance and biomass transfer and of nocturnal insects migrating above the southern UK and the ECP.

**Measure and region**	**Hu et al. 2016: Southern UK (300 km width)**	**Current study: ECP (600 km width)**	**Difference between UK and China**
Nocturnal overflight (km^{-1}) (billions)			
Larger Insects	0.0108	0.394	36.5× i
Small Insects	3.01	15.1 (14.2~16.1)	5.04× (4.73~5.35)
Total	3.02	15.5 (14.6~16.5)	5.15× (4.84~5.45)
Total nocturnal overflight over study area (billions)			
Larger Insects	3.24	237	73.0×
Small Insects	902	9,100 (8,500~9,600)	10.1× (9.45~10.7)
Total	905	9,300 (8,700~9,800)	10.3× (9.68~10.9)
Nocturnal biomass transfer (t km^{-1})			
Larger Insects	0.716	12.7	17.8×
Small Insects	2.32	11.7 (10.9~12.4)	5.02× (4.72~5.33)
Total	3.04	24.4 (23.7~25.1)	8.03× (7.80~8.27)
Total nocturnal biomass transfer over study area (t)			
Larger Insects	215	7,600	35.5×
Small Insects	696	6,990 (6,500~7,400)	10.1× (9.43~10.7)
Total	911	14,600 (14,200~15,000)	16.1× (15.6~16.5)
